# Characterization of University Hazing in 74 Brazilian Medical Schools and the Development and Validation of the University Hazing Self-Report Scale (UNI-Hazing)

**DOI:** 10.1192/j.eurpsy.2025.1820

**Published:** 2025-08-26

**Authors:** L. Fernandes Berto, R. Furlan Damiano, G. Lucchetti, E. Constantino Miguel, H. Vallada, L. F. Dilalla, A. Lamas Granero Lucchetti, E. J. Allan, D. Kerschner, J. Piasseschi de Bernardin Gonçalves, B. Besteti Fernandes Damiano

**Affiliations:** 1 University of São Paulo, São Paulo, Brazil; 2 Department of Psychiatry, University of São Paulo, São Paulo; 3 Federal University of Juiz de Fora, Juiz de Fora, Brazil; 4 Southern Illinois University, Illinois; 5 University of Maine, Orono, United States

## Abstract

**Introduction:**

University hazing is a common practice that impacts students’ mental health and well-being, especially in medical schools. Despite its common occurrence, there is a lack of reliable tools to assess hazing experiences and perceptions among students.

**Objectives:**

This study aimed to evaluate hazing experiences, attitudes, and impacts and to develop and validate the University Hazing Self-Report Scale (UNI-Hazing) among Brazilian medical students.

**Methods:**

This was a cross-sectional study conducted among Brazilian medical students. The UNI-Hazing scale was developed as a four-part questionnaire designed to assess personal experiences with hazing, students’ opinions, and its perceived impact on their well-being. Participants also completed a sociodemographic questionnaire and established scales including the Johns Hopkins Learning Environment Scale (JHLES), the Medical Student Stress Factor Scale (MSSF), the Generalized Anxiety Disorder 7-item (GAD-7), and the Patient Health Questionnaire-9 (PHQ-9). We conducted exploratory factor analysis to uncover latent factors and assessed internal consistency, test-retest reliability, and convergent validity.

**Results:**

1,017 medical students from 74 universities across Brazil participated in the study. While the majority of students did not report being victims or witnesses of hazing, certain hazing behaviors, such as body painting and forced solicitation for money, were relatively common. Hazing incidents most frequently occurred at parties, followed by sports associations and fraternities, with fewer on-campus incidents. Students largely held negative views on hazing. Factor analysis revealed three subscales within UNI-Hazing: “Social Pressure and Institutional Responsibility”, “Emotional Harm and Ethical Concerns” and “Physical Hazing and Power Dynamics”. The scale demonstrated strong internal consistency (Cronbach’s alpha: 0.93, 95% CI: 0.92–0.93) and test-retest reliability (Pearson correlation coefficients: 0.44–0.84). Correlations with the external scales supported the scale’s validity, showing positive correlations with MSSF, GAD-7, and PHQ-9, reflecting the psychological impacts of hazing, and negative correlations with JHLES, indicating that as hazing experiences increase, positive life experiences may decrease.

**Conclusions:**

The UNI-Hazing scale is a reliable and valid measure for assessing hazing experiences and perceptions among medical students in Brazil. The findings highlight the need for universities to implement anti-hazing policies and support systems for students impacted by hazing practices.

**Disclosure of Interest:**

None Declared

